# Couples' voluntary counselling and testing and nevirapine use in antenatal clinics in two African capitals: a prospective cohort study

**DOI:** 10.1186/1758-2652-13-10

**Published:** 2010-03-15

**Authors:** Martha Conkling, Erin L Shutes, Etienne Karita, Elwyn Chomba, Amanda Tichacek, Moses Sinkala, Bellington Vwalika, Melissa Iwanowski, Susan A Allen

**Affiliations:** 1 Rwanda Zambia HIV Research Group, Emory University, 1520 Clifton Rd NE, Rm 235, Atlanta, Georgia, USA; 2 Department of Pathology, School of Medicine and Department of Global Health, School of Public Health, Emory University, 1520 Clifton Rd NE, Rm 235, Atlanta, Georgia, USA; 3 University Teaching Hospital and University of Zambia School of Medicine, Lusaka, Zambia; 4 Catholic Medical Mission Board, PO Box 320146, Woodlands Main, Lusaka, Zambia; 5 Children's Hospital of Philadelphia, 34th Street and Civic Center Boulevard, Philadelphia, USA

## Abstract

**Background:**

With the accessibility of prevention of mother to child transmission (PMTCT) services in sub-Saharan Africa, more women are being tested for HIV in antenatal care settings. Involving partners in the counselling and testing process could help prevent horizontal and vertical transmission of HIV. This study was conducted to assess the feasibility of couples' voluntary counseling and testing (CVCT) in antenatal care and to measure compliance with PMTCT.

**Methods:**

A prospective cohort study was conducted over eight months at two public antenatal clinics in Kigali, Rwanda, and Lusaka, Zambia. A convenience sample of 3625 pregnant women was enrolled. Of these, 1054 women were lost to follow up. The intervention consisted of same-day individual voluntary counselling and testing (VCT) and weekend CVCT; HIV-positive participants received nevirapine tablets. In Kigali, nevirapine syrup was provided in the labour and delivery ward; in Lusaka, nevirapine syrup was supplied in pre-measured single-dose syringes. The main outcome measures were nurse midwife-recorded deliveries and reported nevirapine use.

**Results:**

In eight months, 1940 women enrolled in Kigali (984 VCT, 956 CVCT) and 1685 women enrolled in Lusaka (1022 VCT, 663 CVCT). HIV prevalence was 14% in Kigali, and 27% in Lusaka. Loss to follow up was more common in Kigali than Lusaka (33% vs. 24%, p = 0.000). In Lusaka, HIV-positive and HIV-negative women had significantly different loss-to-follow-up rates (30% vs. 22%, p = 0.002). CVCT was associated with reduced loss to follow up: in Kigali, 31% of couples versus 36% of women testing alone (p = 0.011); and in Lusaka, 22% of couples versus 25% of women testing alone (p = 0.137). Among HIV-positive women with follow up, CVCT had no impact on nevirapine use (86-89% in Kigali; 78-79% in Lusaka).

**Conclusions:**

Weekend CVCT, though new, was feasible in both capital cities. The beneficial impact of CVCT on loss to follow up was significant, while nevirapine compliance was similar in women tested alone or with their partners. Pre-measured nevirapine syrup syringes provided flexibility to HIV-positive mothers in Lusaka, but may have contributed to study loss to follow up. These two prevention interventions remain a challenge, with CVCT still operating without supportive government policy in Zambia.

## Background

Twenty years of research in Africa confirm that couples' voluntary counselling and testing (CVCT) is an effective, feasible and popular HIV prevention intervention in the largest at-risk population in the world, African couples [1-6]. The majority of the estimated 22.5 million people living with HIV infection in sub-Saharan Africa are married and of reproductive age [7], and most new infections are acquired from spouses [8,9].

In parallel with the high prevalence of HIV in women, sub-Saharan Africa also represents 90% of global mother to child transmissions [7]. Nevirapine (NVP), an easily administered and cost-effective antiretroviral drug, reduces mother to child transmission by up to 50% [10-16]. As access to NVP improves, the expansion of individual voluntary counselling and testing (VCT) services in antenatal care has become a priority in high-prevalence, low-resource areas. Providing VCT to women attending antenatal care clinics, along with targeted provision of NVP, has been tested and shown to be a cost-effective intervention to reduce vertical transmission of HIV [10-14,17-19].

VCT has proven to be feasible and acceptable in antenatal clinics throughout Africa and the strides made in increasing the availability of prevention of mother to child transmission (PMTCT) to pregnant women are encouraging [14,17,20]. However, VCT for partners, a critical component in the prevention of HIV in both mother and child, is not as readily available and remains a missing link in 2010. CVCT has been shown to reduce HIV risk among couples [2,21-23], and partner participation has been suggested as a potential factor to leverage PMTCT programmes [24-29]. Given that the majority of antenatal patients have partners, offering CVCT at antenatal clinics, along with providing PMTCT services to the HIV-positive women, could be a solution [26]. The combination of the two interventions would mutually reinforce prevention of horizontal and vertical transmission.

To explore the feasibility of establishing CVCT in antenatal clinics, we trained clinic staff at two antenatal clinics in Lusaka, Zambia, and two antenatal clinics in Kigali, Rwanda, to provide same-day rapid VCT, CVCT and appropriate NVP for PMTCT [30]. We hypothesized that CVCT would improve follow up and adherence with the PMTCT-NVP regimen.

## Methods

### Setting and population

The capital cities of Rwanda in east-central Africa and Zambia in south-central Africa were the locations for this study. The Rwanda Zambia HIV Research Group (RZHRG) had been established in these cities in 1986 and 1994, respectively. Rwanda and Zambia have populations with similar economic constraints, but with different health systems for the delivery of infants and the provision of nevirapine.

A comparative analysis of these differences was undertaken to learn more about effective methods of introducing counselling and testing for HIV in antenatal clinics, the impact of involving a woman's partner in counselling, and the use of nevirapine by HIV-positive women, depending on the method used for its distribution. In 2001, pregnant women in Kigali, Rwanda, received antenatal care at government clinics located throughout the city and delivered at the centrally located *Centre Hospitalier de Kigali*. In Lusaka, Zambia, pregnant women typically delivered at the same clinic where they received antenatal care, with complicated cases referred to the University Teaching Hospital.

Two high-volume antenatal clinics were selected in each capital city. All research activities were conducted by RZHRG in collaboration with local government authorities, the Ministry of Health Treatment and Research AIDS Center (TRAC) in Kigali, and the Ministry of Health Counseling Unit and District Health Clinics in Lusaka. The study and informed consent documents were reviewed and approved by the Institutional Review Boards in the US (OHRP IRB 196), Rwanda (OHRP IRB 1497) and Zambia (OHRP IRB 1131).

### Procedures

At the time this study was initiated (2001), HIV testing was not yet available in government clinics in Kigali and Lusaka. Experienced counsellor trainers from RZHRG provided clinic staff with didactic and practical training in VCT, CVCT and PMTCT [31]. This prospective cohort study included two possible treatments over the time the women were in the study, i.e., counselling and testing for HIV and, for HIV-positive women, nevirapine to take at the beginning of labour. The major source of bias was expected to be loss to follow up, an outcome analyzed by this paper.

**Figure 1:**
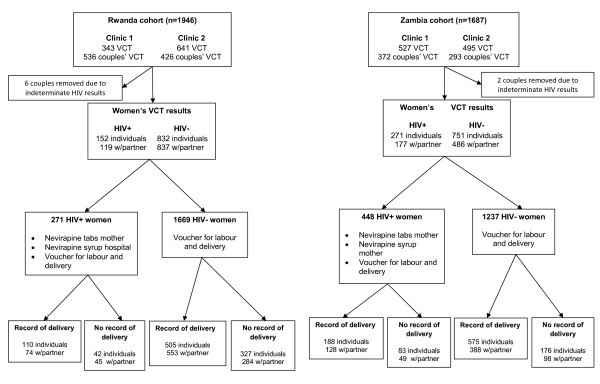
**Screening, recruitment and follow up of volunteers at antenatal clinics in Rwanda and Zambia**.

Between March and December 2001, a convenience sample of pregnant women was recruited from among those seeking antenatal care at the four clinics (Figure 1). Couples were recruited from the sample when the women chose to attend CVCT with their partners. This method of sampling was used in order to study the population of interest in this clinical setting. Exclusion criteria included: known or suspected pregnancy complications (multiple gestation, pregnancy-induced hypertension, diabetes mellitus, anemia, significant third trimester bleeding, premature rupture of membranes, or known or suspected fetal anomaly); known or suspected allergy to nevirapine; and expressed desire to deliver at a non-participating clinic or hospital.

Women presenting for routine antenatal care at a study clinic were invited to participate in the same-day VCT programme. The weekday VCT programme could accommodate only 10 to 15 women per day out of the 30 to 80 women seeking antenatal care each day. For ethical reasons, this limited capacity dictated that priority for testing be given to those in advanced gestation. All antenatal visitors, whether they had tested for HIV or not, received written invitations for weekend CVCT services that were provided at the same facility. Women who were not tested could come in on the weekend with their partner or wait until their next ANC visit for individual testing.

VCT and CVCT services began in the morning with a group discussion, followed by individual and/or couple pre-test counselling, written informed consent, and phlebotomy. Same-day post-test counselling of HIV was received after lunch (provided by the study). The CVCT model used was developed initially in Rwanda in 1988 and had been implemented in Zambia by the RZHRG since 1994 [6].

Participants were given a delivery voucher for prepayment of labour and delivery expenses. At the post-test counselling session, all HIV-positive women, in both cities, were given a tablet containing 200 mg of NVP, which they were instructed to take at the onset of labour. Women in Lusaka also received a delivery pack with gloves, a cord clamp and pads. The central hospital, which is the only delivery facility in Kigali, was stocked with NVP syrup (Manheim Ingleheim-GMBH) to administer to newborns at delivery. In Lusaka, women could deliver at any of the Lusaka district health facilities; as none were yet stocked with NVP, women were given a pre-filled syringe of NVP syrup with instructions to administer to their infants as soon as possible after birth. Extra pre-filled syringes were provided to the two Lusaka clinics participating in the study and to the University Teaching Hospital.

### Laboratory procedures

Same-day HIV testing was conducted using a two-step rapid HIV test algorithm [30], Determine HIV-1/2 test (Abbott Laboratories, Belgium) for screening and the Capillus HIV-1/HIV-2 test (Trinity Biotech Ltd, Ireland) as the confirmatory test. Quality control of doubtful or discrepant samples and 10% of routine samples was performed with a two-ELISA algorithm at a reference laboratory in each city.

### Data collection and analysis

Study staff recorded demographic information and obstetric history during pre-test counselling. HIV results were recorded in a laboratory log coded by study ID. Follow-up data was collected at delivery, using the delivery payment vouchers provided to all women during post-test counselling. Compliance of HIV-positive mothers with the NVP tablet was assessed by self-report in the labour and delivery ward. Labour and delivery nurses also recorded the time of administration of NVP to the newborns. Data from women who delivered at a clinic other than their antenatal clinic were captured when they came for post-partum and newborn checkups, with follow up completed by December 2002.

Univariate statistics were calculated for the demographic variables; numbers and percents for categorical variables and means with standard deviations for continuous variables. HIV tests were analyzed to yield proportions of the study population that were infected, depending on whether they had undergone VCT or CVCT. Bivariate analysis was conducted between the outcome variables of interest, i.e., having a record of delivery and whether nevirapine was used or not and the variables found in Table 1. The first set of bivariate statistics were calculated for all of the women in the study that had information on the variables chosen (n = 3316) and then for the subgroup of HIV-positive women (n = 654). Pearson's chi-square statistics were used to determine statistical significance at p < 0.05 for categorical variables while t-tests were calculated for continuous variables.

**Table 1:** Demographic characteristics of antenatal women and couples in Kigali, Rwanda and Lusaka, Zambia (n = 3625)

	Kigali			Lusaka		
	**Individuals** **n = 984** **mean (SD)**	**Couples** **n = 956** **mean (SD)**	**Total** **n = 1940** **mean (SD)**	**Individuals** **n = 1022** **mean (SD)**	**Couples** **n = 663** **mean (SD)**	**Total** **n = 1685** **mean (SD)**

Age						
Man		32.0 (7.8)	32.0 (7.8)		30.5 (6.8)	30.5 (6.8)
Woman	25.8 (5.6)	26.1 (5.4)	25.9 (5.5)	24.3 (5.7)	24.1 (5.4)	24.3 (5.6)

Years cohabiting*	4.9 (4.8)	4.5 (4.5)	4.7 (4.7)	6.0 (5.4)	5.2 (4.8)	5.7 (5.1)

Years living in						
Kigali/Lusaka	4.2 (4.7)	3.8 (4.1)	3.9 (4.4)	5.2 (5.2)	4.2 (4.1)	4.8 (4.8)

Prior pregnancies	1.9 (1.9)	2.0 (1.9)	2.0 (1.9)	2.0 (1.9)	2.0 (1.8)	2.0 (1.9)

	%	%	%	%	%	%

Marital status						
Legal	25	33	29	9	14	11
Traditional	2	5	3	61	64	62
Common law	53	62	58	21	22	22
Single/widow	20	0	10	9	0	5

Children in the home						
Couple's						
None	48	42	45	44	39	42
≥1 child	52	58	55	56	61	58
Man's						
None	92	93	93	87	90	88
≥1 child	8	7	7	13	10	12
Woman's						
None	86	93	89	89	88	89
≥1 child	14	7	11	11	12	11
Orphan/other						
None	81	78	80	76	80	78
≥1 child	19	22	20	24	20	22

Difficult previous pregnancy	20	22	21	13	13	13

Previous HIV test	21	30	26	3	8	5

*For those with spouses

Models were formed for multivariate analysis from variables that were statistically significant in the bivariate analysis or were considered important in the context of the study. Odds ratios and confidence intervals were derived for the variables in the multivariate models. The likelihood ratio X^2 ^was calculated to illustrate whether the predictors used in the model were helpful in interpreting the outcome variable. This analysis was conducted using Stata 10 statistical software [32].

## Results

### Demographics

A total of 3633 women received VCT in the four antenatal clinics in Lusaka and Kigali. Eight couples, two from Kigali and six from Lusaka, were not included in this analysis due to discrepant HIV rapid test results for one of the partners (Figure 1). Of the 3625 women remaining, 1619 received CVCT (956 in Kigali and 663 in Lusaka), and 2006 women were tested alone (984 in Kigali and 1022 in Lusaka) (Table 1).

Demographically, the study population was broadly homogenous across cities and among women who tested alone versus those who tested with their partners. The mean age of women was two years older in Kigali (26 years) than Lusaka (24 years); among male partners tested, mean age in Kigali was 32 compared with 31 in Lusaka. Women in both countries had a mean of two previous pregnancies and had been cohabiting with their partners for five years in Kigali and six years in Lusaka (Table 1).

Marriages between Kigali participants, whether they tested alone or with their partners, were likely to be common law unions (58%) or legal marriages (29%), whereas Lusaka women more commonly married traditionally (62%) or in common law unions (22%) (Table 1). The number of Kigali and Lusaka women tested alone who reported being "single" (193/984 [20%] vs. 91/1022 [9%]) was significantly different (p = 0.000) when compared with other marriage states in Kigali and Lusaka, possibly because of the attendance of genocide widows in the Kigali clinics.

The number of women reporting children living in the home was also significantly different (p = 0.000) between Kigali (69%) and Lusaka (76%) (not shown). Most children were those of the woman and her current spouse, with 7% to 12% of households including children of the man or the woman with other partners. Twenty percent of households in Kigali and 22% of households in Lusaka included orphans or children who were not biological children of the pregnant woman and/or her current spouse.

Reported cases of a difficult previous pregnancy, defined as either a spontaneous abortion/miscarriage or a stillbirth/child death within two days of birth, was also significantly different (X^2 ^= 40.949, p = 0.000) between the capitals (403/1940 [21%] in Kigali vs. 215/1685 [13%] in Lusaka).

### HIV prevalence and HIV testing history

The prevalence of HIV was significantly higher among women in Lusaka (448/1685, or 27%) than in Kigali (271/1940, or 14%) (X^2 ^= 90.30, p = 0.000). Table 2 shows HIV prevalence and NVP use stratified by whether women were single, and if married, whether they tested alone or with their spouses.

**Table 2:** Retention of women and their NVP use if HIV-positive in Kigali (n = 1940) and Lusaka (n = 1685)

	Single		Married tested alone		Couples tested				
	HIV+	HIV-	HIV+	HIV-	M+F+	M-F+	M+F-	M-F-	Total
**Kigali ** (n = 1940)	% (n = 193)		% (n = 791)		% (n = 956)				% (n = 1940)

No record of delivery	24	34	30	38	35	41	36	29	33

Record of delivery		66		62			64	71	57
With NVP	66		60		59	52			9
No NVP	10		10		6	7			1

**Lusaka ** (n = 1685)	% (n = 91)		% (n = 931)		% (n = 663)				% (n = 1685)

No record of delivery	40	15	30	24	27	26	29	19	24

Record of delivery		85		76			71	81	57
With NVP	50		55		58	54			15
No NVP	10		15		15	20			4

The HIV prevalence in Lusaka was 22% for single women and 27% for both married women tested with their partners and those who tested alone. In Kigali, single women had a higher prevalence of HIV (21%) than married women tested alone (14%) or with their partners (13%). Women in Kigali were more likely to have been previously tested for HIV (498/1940, or 26%) than women in Lusaka (83/1685, or 5%) (X^2 ^= 288.33, p = 0.000) (Table 1).

Among couples tested in Kigali (n = 956), 8% were concordant HIV positive, 9% were HIV discordant (Table 2) and the gender of the positive partner in the discordant couples was equal, with 42 couples having HIV-positive men and HIV-negative women, and 42 couples having HIV-negative men and HIV-positive women. In Lusaka, where 663 couples tested, 19% of couples were concordant HIV positive and 17% were HIV discordant; the frequency of the HIV-positive partner in the discordant couples was again equally distributed.

### Delivery

In Rwanda, of those with records of delivery, 92% of women delivered at the Central Hospital, while in Zambia, 83% of women delivered in the same facility that had provided their antenatal care (Table 3). There were no significant differences in delivery location between HIV-positive and HIV-negative women and whether the partner was tested (not shown).

**Table 3:** Delivery location of mothers in Kigali and Lusaka as determined by vouchers collected by nurse midwives

	Kigali (n = 1292)		Lusaka (n = 1279)	
	n	%	n	%
Same clinic as ANC	0	0	1066	83
Hospital	1184	92	77	6
Other health facility	9	1	25	2
Home/dwelling	73	6	71	6
Other	21	2	29	2
Spontaneous abortion/stillbirth	5	0	11	1

Having no record of delivery, i.e., the woman did not present a study voucher at the time of delivery, was defined as "lost to follow up" (LFU) for this study. Of all women participating in the study, 29% were LFU. In Kigali, 648/1940 (33%) women were LFU, while in Lusaka, 406/1685 (24%) were LFU (X^2 ^= 37.88, p = 0.000) (Table 2). Of those women lost to follow up, 13% (87/648) were HIV positive in Kigali, and 33% (132/406) were HIV positive in Lusaka. In Kigali, there was no difference between HIV-positive and HIV-negative women who were LFU (32% vs. 34%, p = 0.625), while in Lusaka, HIV-positive and HIV-negative women had significantly different LFU (30% vs. 22%, X^2 ^= 0.239, p = 0.002).

Broadly, across both cities, 27% (440/1619) of those LFU were tested as couples and 31% (614/2006) were tested alone (Table 2). When stratified by city, this trend remained, i.e., women tested with their partners were less likely to be LFU than those tested alone. Of those women in Kigali, loss to follow up was 31% (293/956) in CVCT and 36% (355/984) in VCT (X^2 ^= 6.424, p = 0.011); in Lusaka, loss to follow up was 22% (147/663) in CVCT versus 25% (259/1022) in women tested alone (X^2 ^= 2.21, p = 0.137).

Multiple logistic regressions were performed to determine predictors of women having a record of delivery (Table 4). Models were formed for all cohabiting women in the study and for cohabiting women who were HIV positive. Among women with cohabiting partners, variables independently predictive of having a record of labour and delivery (p-value < 0.05) were: (1) testing with the partner (OR = 1.28 [CI 1.100, 1.494]); (2) residing in Lusaka (OR = 1.73 [CI 1.473, 2.034]); and (3) having orphans or other non-biological children living in the home (OR = 1.24 [1.016, 1.509]). This means that, when controlling for the other factors in this model, the odds of a woman having a record of delivery were 28% more likely for a woman counselled and tested with her partner (p = 0.001) than for a woman who tested alone.

**Table 4:** Predictors of having a record of delivery in the subsets of all of the women and the HIV-positive women, as determined by logistic regression models

	All women** (n = 3316)		HIV+ women** (n = 654)	
Variables	Odds ratio (CI)	p-value	Odds ratio (CI)	p-value
Woman's increasing age*	1.006 (0.990, 1.021)	0.485	1.046 (1.007, 1.087)	0.021
HIV status of woman (HIV+ = 1)	0.832 (0.687, 1.009)	0.061		
Partner tested* (yes = 1)	1.282 (1.100, 1.494)	0.001	0.974 (0.692, 1.370)	0.879
Capital city* (Lusaka = 1)	1.731 (1.473, 2.034)	0.000	1.489 (1.025, 2.165)	0.037
Difficult previous pregnancy* (yes = 1)	1.152 (0.938, 1.415)	0.177	1.606 (0.997, 2.584)	0.051
Children living in the home (≥1 = 1)	0.888 (0.727, 1.084)	0.243	0.669 (0.425, 1.054)	0.083
Orphans living in the home* (≥1 = 1)	1.238 (1.016, 1.509)	0.035	1.463 (0.931, 2.301)	0.099
Likelihood ratio χ^2^	56.52	0.000	16.87	0.009

*Significant in bivariate analysis (p < 0.05)

**Excluding single women

Also, women with partners in Lusaka were 73% more likely to have a record of delivery than those living in Kigali, considering all other variables (p = 0.000). Women living in households with orphans or other non-biological children were 24% more likely to have a record of delivery (p = 0.035). Age, HIV status, reporting a difficult previous delivery, and other children in the home were not significant predictors of a record of delivery in this model.

In the logistic regression model for HIV-positive women with cohabiting partners, predictors of a record of delivery included: (1) woman's older age (OR 1.05 [1.007, 1.087]); (2) living in Lusaka (OR 1.49 [1.025, 2.165]); and (3) a difficult previous pregnancy (OR 1.61 [0.997, 2.584]). In this population, HIV-positive women with partners were 5% more likely to have a record of delivery with each year they gained in age (p = 0.021) and 49% more likely to have a record of delivery if they lived in Lusaka (p = 0.037) instead of Kigali. Having a difficult previous pregnancy meant that HIV-positive women were 61% more likely to have a record of delivery than those who had not had a difficult pregnancy (p = 0.051). Having a partner tested, and any children and/or orphans living in the couple's home were not significant predictors of having a record of delivery in this model (Table 4).

### Nevirapine compliance

Nevirapine use in this study was recorded if the mother, infant or both took the recommended dose at the appropriate time. Overall, of the women who were HIV positive and had a record of delivery, 82% achieved some NVP use (162/185, or 88%, in Rwanda vs. 251/319, or 79%, in Zambia; p = 0.012). Multiple logistic regression models of NVP compliance were not significant; nor were any of the covariates.

## Discussion

Couples' voluntary counselling and testing offered on the weekends in high-volume antenatal clinics was feasible, and when partners participated together in counselling and testing, women were more likely to present a study voucher at the time of delivery in both Kigali and Lusaka. In this study, carried out in 2001, CVCT helped prevent a loss to follow up, which meant more appropriate care was given during the delivery.

We found in Lusaka, where the NVP tablet and syrup were provided to pregnant women at post-test counselling sessions, that HIV-positive women were less likely to have a record of delivery than HIV-negative women. In contrast, in Kigali, where NVP syrup was available only in the labour and delivery ward, HIV-positive women were more likely to have a record of labour and delivery. Partner participation was not associated with differences in NVP use.

At the time of study conception (2000), VCT was not yet established in antenatal clinics in Kigali and Lusaka and CVCT had been implemented only in a few specialized research centres. VCT is now, as a matter of policy, offered in antenatal clinics in both Rwanda and Zambia. Antenatal CVCT, which provides an important opportunity to identify and reduce transmission of HIV among discordant couples, has been more slowly adopted, if at all. Since this study took place, research indicates that promoting CVCT in the community and inviting couples together increases the uptake of couples' counselling [22].

Once discordancy is established through CVCT, special attention needs to be paid to couples where the woman is the HIV-positive partner. It has been shown, both here and in other studies, that couples where the woman is the positive partner are more often lost to follow up than couples where the man is the positive partner [33,34]. HIV-positive women need more counselling and psychosocial support as they approach delivery in order to disclose their status and protect themselves and their infants [35]. HIV-negative women in a discordant couple also need to be monitored so that, in the event that they become HIV infected as they near delivery, they and their infants can be provided with NVP.

The initial study design was to randomize women to VCT or CVCT, but, for ethical reasons, this changed. Though logistically practical, this method of service delivery and modification to the study design may have biased the study analysis. Those women who were able to attend CVCT with their partners may have been unique, i.e., their partners were willing to participate, when compared with those women who underwent VCT.

Also, the outcomes of interest, having a record of delivery and taking nevirapine at the appropriate time were subject to a loss-to-follow-up bias. However, the order of treatment delivery was the same in each city for all study participants, thus avoiding any "order" effects that could have influenced the outcome. The HIV prevalence findings of 14% in Kigali and 27% in Lusaka among antenatal clinic attendees in this study are consistent with published prevalence data for that time in both capital cities [7,36].

A substantial proportion (33% in Kigali and 24% in Lusaka) of study participants were without a record of delivery (LFU). The follow-up rates were different between the two cities, indicating that delivery practices between cities may have impacted study findings. While these percentages seem high, Manzi *et al*, who conducted an antenatal VCT/PMTCT study in Malawi in 2002/03, experienced a loss to follow up of 68% by the time of delivery, suggesting that this was not abnormally high for this intervention at the time [37].

There was no difference in LFU between HIV-positive and HIV-negative women in Kigali. In 2001, 21% of the women in Kigali who came to the antenatal clinics participating in this study already knew their HIV status, perhaps diminishing the impact of VCT among this population. The significant difference in loss to follow up between women tested alone (36%) and those tested with their partners (31%) highlights the importance of partner participation not only in CVCT, but also in helping his partner to identify herself at delivery, enhancing PMTCT. In Lusaka, there was a significant difference in loss to follow up between HIV-positive and HIV-negative women. Here, the availability of NVP for both mother and infant contributed to more loss to follow up since those mothers did not have to disclose their status in order to protect themselves and their infants.

Fewer participants were lost to follow up when they delivered in the clinic where they received antenatal care and VCT, a place where their HIV status was already known, as was the case in Lusaka. In Kigali, where women delivered in the hospital, a different facility with different providers than their antenatal service providers, there were more HIV-positive women without a record of delivery.

Taking nevirapine meant disclosing the woman's HIV status, creating a risk of being stigmatized. PMTCT programmes utilizing single-dose NVP regimens may consider providing NVP syrup to mothers to administer to infants themselves [11] in order to protect infants when a mother is too fearful to disclose her status. Self-report of NVP use was a limitation of this study. However, in another study of self-reported adherence to NVP in Kenya, 90% of women reported taking their dose of NVP [38], similar to the 79% to 88% reported here.

In routine service delivery, there was potentially less incentive to self-identify since the study provided the delivery fee in return for the follow-up information. However, women who delivered at home or in a different facility would not need the vouchers, and women who preferred to keep their serostatus private may have preferred to pay labour and delivery costs rather than produce the voucher. Women in Kigali also faced greater distances to reach the one health service facility available to them for delivery.

## Conclusions

Although a limitation of this study was the "newness" of PMTCT, VCT and CVCT in both populations, uptake of NVP remains a problem throughout Africa [39]. Both Rwanda and Zambia have made efforts to incorporate PMTCT into routine maternal health services [40], but success in NVP delivery remains difficult to implement and measure [39].

At the time of this writing, eight years after the study presented here, partner testing has been integrated into routine services in Kigali, where more than 80% of pregnant women are now tested with partners (unpublished data, TRAC-Plus, Ministry of Health, Rwanda). In Lusaka, however, partner testing remains limited to weekends, and less than 10% of pregnant women are tested with partners (unpublished data, RZHRG). Incentives, i.e., lunch, transport reimbursement and childcare, are still offered to increase the uptake of CVCT in Lusaka. A low level of male involvement remains a factor in determining effective HIV testing and PMTCT service delivery and acceptability [35,40-43].

Prevention of transmission between partners in discordant relationships and from an HIV-positive mother to her infant remains a critical factor in controlling the spread of HIV in sub-Saharan Africa [9]. Additional research is necessary to explore the effect of partner participation in antenatal CVCT. The combination of HIV prevention through PMTCT regimen compliance, increased contraception counselling with couples, and reduced risk behaviour among discordant couples could impact transmission of HIV to family members.

Our finding that NVP uptake was not enhanced by partner participation in CVCT needs to be further explored, particularly in Kigali, where CVCT is now a norm. Partner participation may also be important to PMTCT programmes utilizing highly active antiretroviral treatment regimens as more countries become able to implement World Health Organization recommendations [44]. Prolonged regimens are more difficult to protect from unintentional disclosure, and compliance may benefit with partner support [45], confirming the importance of a comprehensive family approach to HIV prevention in urban sub-Saharan Africa.

## Competing interests

The authors declare that they have no competing interests.

## Authors' contributions

MC led the writing, editing and submission of the article, and the final analysis of the data. ES assisted with this study in Kigali, Rwanda, and Lusaka, Zambia, and conducted the preliminary analyses, writing and literature review. EK is the RZHRG Director in Kigali and directed the study at the Kigali clinics. EC is the Senior Co-Investigator of the Zambia Emory HIV Research Project and oversaw the study activities in Lusaka, Zambia. AT contributed to the data analysis and manuscript preparation. MS was the Director of the Lusaka District Health Management Team in Zambia and facilitated the study conduct and clinic staff availability in Lusaka. BV assisted with data collection as the Study Physician in Zambia and contributed to the study design and implementation. MI managed the data during the study, contributed to the data analysis, first draft of the Background and Methods sections, and presentation of the data at the IAS conference in Paris. SA, based at Emory University, is the Director of the RZHRG and was the principal investigator for the World AIDS Foundation grant. She wrote the study grant and coordinated the research in both Kigali and Lusaka. She contributed to the preparation of the manuscript, including the final editing. All authors read and approved the final manuscript.
